# Additive Manufacturing of Polymeric Bioresorbable Stents: A Mechanical Performance Perspective

**DOI:** 10.34133/bmr.0259

**Published:** 2026-01-23

**Authors:** Gurminder Singh, Irina Khaydukova, Kevin Walsh, Colin J. McMahon, William Ronan, Eoin D. O’Cearbhaill

**Affiliations:** ^1^ School of Mechanical and Materials Engineering, UCD Centre for Biomedical Engineering, University College Dublin, Dublin, Ireland.; ^2^ Department of Mechanical Engineering, Indian Institute of Technology Bombay, Mumbai, India.; ^3^ Department of Paediatric Cardiology, Children’s Health Ireland at Crumlin, Dublin, Ireland.; ^4^ School of Medicine, University College Dublin, Dublin, Ireland.; ^5^ Biomedical Engineering, University of Galway, Galway, Ireland.

## Abstract

The integration of biomaterials and additive manufacturing (AM) has revolutionized the design, manufacturing, and clinical applications of permanent and bioresorbable implants. AM offers design flexibility and potential for mass customization but poses challenges for scalable manufacturing. Unlike other high-commodity implantable devices that are already clinically approved, stent AM is still in the early phases of research and development. Here, following the recent Food and Drug Administration approval of Abbott’s Esprit stent for below-the-knee use, we examine the current prospects for AM of polymeric stents, specifically focusing on polymeric bioresorbable stent geometry, material composition and mechanical properties, and surface quality, predominantly intended for cardiovascular applications. The advancement of bioresorbable polymeric stents is shown through a comparison with metallic stents commonly used in clinical practice. The different AM techniques used for stent fabrication and the level of currently fabricated bioresorbable stents are reviewed. A road map for translating AM stents from the research laboratory to the clinic is proposed.

## Introduction

Polymeric bioresorbable stent (BRS) technology is at a crossroads [1]. The recent Food and Drug Administration (FDA) approval of Abbott’s Esprit BTK (below the knee) Everolimus Eluting Resorbable Scaffold System has brought renewed optimism toward the prospect that their initial promise can be realized in specific clinical indications and will therefore refocus research and development efforts on material and design optimization [2]. Three-dimensional (3D) printed medical devices are being adopted in many other spheres of Medtech, particularly where there is a focus on patient-specific fabrication or geometries that cannot be easily fabricated using conventional techniques [3]. To understand how BRS are likely to develop in the future, and specifically the role rapid prototyping and additive manufacturing (AM) can play, it is important to examine their development in the context of the evolution of stent design, material selection, and fabrication strategies [4].

This review examines the convergence of BRS material evolution and the emergence of 3D printing techniques for the development of polymeric BRS. Here, we summarize the evolution of the clinical need for BRS, the mechanical basis for the selection of candidate stent materials, and the role of 3D printing technologies in stent research, development, and manufacturing. After analyzing the criteria for mechanical stents and material comparison, we used the documented performance characteristics of clinically used stents to benchmark the mechanical performance of the 3D printed stents. Furthermore, we summarize the existing literature on 3D printed stents, highlighting the advantages and disadvantages of various printing modes, strategies, and material selection. This allows us to compare clinically used non-bioresorbable stents (non-BRSs) with their polymer bioresorbable additively manufactured analogs based on the established criteria and benchmark properties, illustrating gaps and potential opportunities for material and design development from a purely mechanical perspective. Finally, we investigate the emerging trends in additively manufactured polymer BRS that present a future road map for development.

### The evolution of stents

#### Clinical need for stents

Stents are implants that open and support hollow lumens or vessels. They are implemented in various fields, with the most common application being blood vessel revascularization. This solution was introduced as a minimally invasive treatment for vessel occlusion that could maintain the restored lumen diameter post-intervention. Balloon angioplasty alone was first used to push atherosclerotic plaques against the vessel walls and restore blood flow. This technique successfully increases vessel diameter, but it results in early vessel recoil and restenosis problems in 30% to 50% of patients [5], necessitating the introduction of a stent scaffolding architecture to preserve vessel patency.

The first generation of bare metal stents (BMSs) led to prolonged intimal hyperplasia, which resulted in in-stent restenosis [4] To address this issue, drug-eluting stents (DESs) were introduced, typically consisting of metal stents coated with a polymer containing an antiproliferative drug (often sirolimus, novolimus, everolimus, or paclitaxel) that elutes throughout 30–80 days [6]. DES lowered the risk of hyperplasia and target lesion revascularization [7–9]. While BMS and subsequent DES have been largely effective at reducing restenosis, they remain permanent implants long after they have performed their primary function, which can result in long-term complications such as stent fracture [10] and requires the patient to remain on dual anti-platelet therapy [11]. Furthermore, permanent supporting solutions are especially disadvantageous in pediatric surgery, where the mismatch of tissue and implant dimensions progresses with child growth [12].

**Fig. 1.**
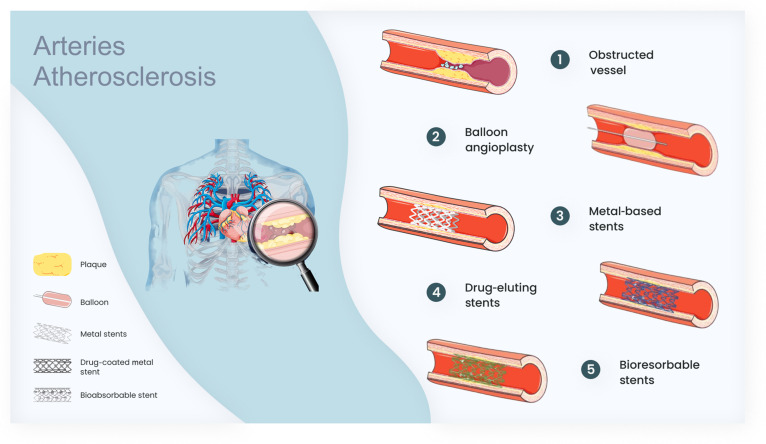
History of stents in the treatment of atherosclerosis, the most commonly deployed endovascular device. Additive manufacturing could enable the next generation of patient-specific stent designs customized for anatomy and lesion type.

Bioresorbable or biodegradable stents have been developed as a solution for the limitations of BMS and DES. BRSs are designed to serve as temporary scaffolds for the vessel wall for several months after implantation [13] and are then completely resorbed, allowing for the restoration of vascular tone when placed within the blood vessel. BRSs can be made from polymers or metals with appropriate mechanical properties and resorption profiles (Fig. 1). BRSs have also been proposed for other applications and deployment sites including the gastrointestinal tract (esophageal, biliary, pancreatic, and duodenal [14–16]), salivary gland [17], ureter [18], prostate [14] trachea [19–23], lacrimal duct [10], and alongside specific vascular applications (anastomosis [24], neurovascular [25], and peripheral [26]). Notably, the Ella-DV stent (ELLA-CS, Czech Republic) is a clinically approved self-expanding BRS, approved for use in patients aged 18 years and older, and is indicated for the management of refractory benign esophageal stenoses**.** The DV stent made from poly(p-dioxanone) (PPDO) presents equivalent results to fully covered self-expandable metal stents [27,28].

Although BRSs are being developed for multiple clinical needs, vascular BRSs remain the most studied application. The first attempt of launching BRS for coronary lesions showed inferior results in short- and mid-term results [29], late luminal loss, and device-oriented composite endpoint due to target vessel myocardial infarction, including peri-procedural myocardial infarction [30]. However, this setback did not stop the development of tens of different BRSs for multiple clinical applications [31]. Importantly, the latest BRSs have shown promising results in clinical trials led by the Esprit BTK (Abbot, USA). Their LIFE-BTK study have demonstrated that 48% fewer patients required reintervention at 2 years than after balloon angioplasty [32,33]. Another peripheral vasculature BRS Motiv (Reva Medical, USA) has also presented promising results in a pilot study with clinically driven target lesion revascularization (cd-TLR) 2% and limb salvage rate 95% at 2 years [34]. Another recent trial reported that the Magnitude peripheral BRS (R3 Vascular, USA) reduced 80.1% diameter stenosis before treatment to 23.0% at 6 months [35].

The BRS Magmaris (Biotronik, Germany) in a prospective, multicenter, first-in-man trial showed target lesion failure defined as a composite of cardiac death, target-vessel myocardial infarction (MI), and cd-TLR at 5.9% and 0% thrombosis in coronary arteries at 2 years. An earlier study of MeRes100 BRS revealed a cumulative major adverse cardiac event rate of 1.87% and in-scaffold late lumen loss of 0.24 ± 0.34 mm [36]. In a study that evaluated Bioheart BRS compared to cobalt-chromium everolimus-eluting stent (CoCr-EES) [37], the in-segment late loss was 0.17 ± 0.38 mm, which was noninferior to CoCr-EES, and clinical outcomes at 3 years were similar in the 2 groups, as were the rates of target lesion failure.

Despite advances in BRS, stents are still almost entirely produced from non-BRS materials. This is due to several factors, including the relative clinical success of DES and the withdrawal from the market of the first clinically trialed BRS, Absorb. Since this study, 480 biomedical companies have proposed a new BRS [38], and Amaranth Medical, Inc. [39] and Reva Medical Inc. [40] have continued their clinical trials. All the varieties of BRS are discussed in detail in Ref. [41]. Nevertheless, the existing recommendations of the Task Force of the European Society of Cardiology state that BRSs should not be preferred over conventional DESs in clinical practice. All devices with CE-mark approval should be planned for a large-scale randomized clinical trial with a planned long-term follow-up [42]. Here, the Absorb stent properties were chosen as the benchmark owing to the predominant number of clinical trials [43–46] and finite element analysis investigations [47–54].

#### Evolution of stents: Fabrication techniques

Irrespective of the type of stent (BMS, DES, or BRS), the stent fabrication processes strongly influence stent mechanical and functional performance [55]. Post-processing can also either boost or weaken the material’s properties.

The traditional available methods for manufacturing stents can be either subtractive (laser cutting) or additive (braiding). More recently, injection molding and 3D printing-based AM have been proposed as alternative fabrication methods, particularly for polymeric BRS. Metal and polymer stents are typically made by micro laser cutting a tube. Materials are often extruded into hypotubes and further laser cut as per the required design [56,57]. Stent laser cutting offers excellent accuracy and precision, but the surface of the stents can be degraded, and a heat-affected region may develop during the cutting process [58,59]. Stents are typically subjected to post-processing thermal cycles and polishing processes to relieve stress concentrations and improve the surface finish.

Braiding involves the wrapping of polymer/metal wires onto a mandrel in controlled orientations [38]. The braiding procedure is used to make endovascular stents of exceptional flexibility and shape recoverability, making it particularly suited for self-expanding stent applications. This type of stent has quite low radial strength and high degrees of foreshortening. However, it is well suited for a range of applications, such as iliac arteries, venous, biliary, tracheobronchial, and shunts [60]. Micro-injection molding is an exploratory method for mass-producing stents to obtain high accuracy and acceptable surface quality. The polymer melts are poured into a mold with a pre-set stent shape, solidified in the mold by cooling, and afterwards removed for post-processing [61,62]. However, due to material filling and demolding issues, injection molding can be limited in manufacturing stents with complex structures and small-scale features.

In the last decade, tremendous advances have been made in adopting AM processes in the biomedical sector. AM is a layer-by-layer manufacturing technique based on a computer-controlled process typically initiated via developing a CAD (computer-aided design) model [63]. The slicing of the tessellated CAD model is performed along with optimized orientation to minimize support materials and build time and selecting processing parameters such as layer thickness, speed, patten shape, infill percentage, and temperature. Further, computer network controlled g-code is generated as a toolpath for specific AM machines. While it remains more economically advantageous to fabricate serial stents by traditional methods, AM provides the opportunity to produce patient-specific stents with varying curvature, thickness, and materials for more complicated lesions based on patient computer tomography (CT) and magnetic resonance imaging (MRI) scans. The success of this strategy has been demonstrated in other medical device sectors, through the widespread adoption of patient-specific orthopedic and dental implants fabricated with the help of AM methods. Moreover, AM methods tend to be much more cost-effective and accessible for research and development purposes. With a sufficient advancement of the technology and the mechanical properties closely mimicking the standard manufacturing techniques, AM could provide the opportunity for stent rapid prototyping and performance exploration.

The fabrication of micrometer-sized products from conventional bioresorbable materials with low porosity and high strength is still challenging for AM. However, it has begun to open up new possibilities in the prototyping and fabrication of stents. For example, using AM, drug-incorporating structures can be manufactured with customized architecture and dosages. Some emerging methods under AM category such as electro writing [64] and 2-photon polymerization [65] have potential for fabricating stents. Despite its promise, substantial material processing and scalability challenges must be overcome, before these devices can secure regulatory approval and progress to clinical use. A recent article [66] summarized the existing AM methods used for vascular stents. However, different stent applications present varying requirements. Therefore, the choice of a suitable AM manufacturing technique is highly dependent on a type of stent. Moreover, not all AM manufacturing methods have been adapted for bioresorbable materials, which further limits their immediate implementation for stents.

Therefore, in the current article, an attempt has been made to review the progress of AM methods for the fabrication of BRS depending on their application. The review of currently proposed AM BRS designs and materials as well as the benchmark properties of non-AM non-BRSs for the main applications contextualize the design goals, which must be achieved via AM methods. These findings allowed us to formulate a road map of future development of AM stents.

## Benchmarking the Performance Characteristics for AM Polymeric BRS

### Comparison criteria for the development of AM polymeric BRS

The main problem associated with the adoption of BRS is the lack of reliable mechanical performance. The stent behavior is usually characterized by the radial strength, crush resistance, and recoil for balloon-expandable stents, while for self-expanding stents, these parameters are replaced with bending stiffness, chronic outward force (COF), and compression resistance. Late thrombotic events, which were the reason for Absorb BVS withdrawal, are associated with low radial strength, which further decreases with progressive degradation of the stent. High elastic recoil is also common in polymer stents [50].

To provide the necessary radial strength, a high elastic modulus material must be chosen. As demonstrated by Bobel et al. [51], to show radial strength close to that of metallic stents, a PLLA stent with Absorb design and strut thickness must have Young’s modulus in the order of 5 GPa. Another important criterion for stent materials is the maximum strength and elongation. A high maximum tensile strength prevents stent fracture during expansion and from the stress of the vascular wall. A high elongation at break is another important criterion because polymeric devices have a lower limit of expansion and can fracture owing to over-dilation. It is important to improve the expandability of BRS while maintaining its radial strength. With lesser strength, BRS requires vessel predilatation and may achieve a lesser acute gain (defined as the difference between pre-procedural minimal luminal diameter [MLD] and immediate post-procedural MLD) than a metallic stent [67].

Another crucial requirement for vascular stents is the minimization of strut thickness. Thinner struts produce less flow disturbance and lower endothelial shear stress gradients [68]. Reduced strut thickness minimizes blood turbulence, which may prevent stent thrombosis. Moreover, thinner struts tend to be more flexible, improving trackability, and have a lower profile, improving crossability compared with conventional second-generation DES. This is a crucial requirement for treating coronary lesions [69]. This requirement tends to favor the use of bioresorbable metals over polymers, at least for small diameter and high radial force requirement applications. The following parameters are also important to take into account when designing a BRS, but they were not analyzed in the current study: degradation time [70], surface smoothness [71], drug loading [72], and printing resolution.

The parameters discussed above are the most important vascular stent requirements and were therefore analyzed in the current study: material (Young’s modulus, maximum stress, and elongation), stent design (bending stiffness, radial strength, compression resistance, and recoil), and strut diameter.

### Potential implementation of AM for BRS

**Fig. 2.**
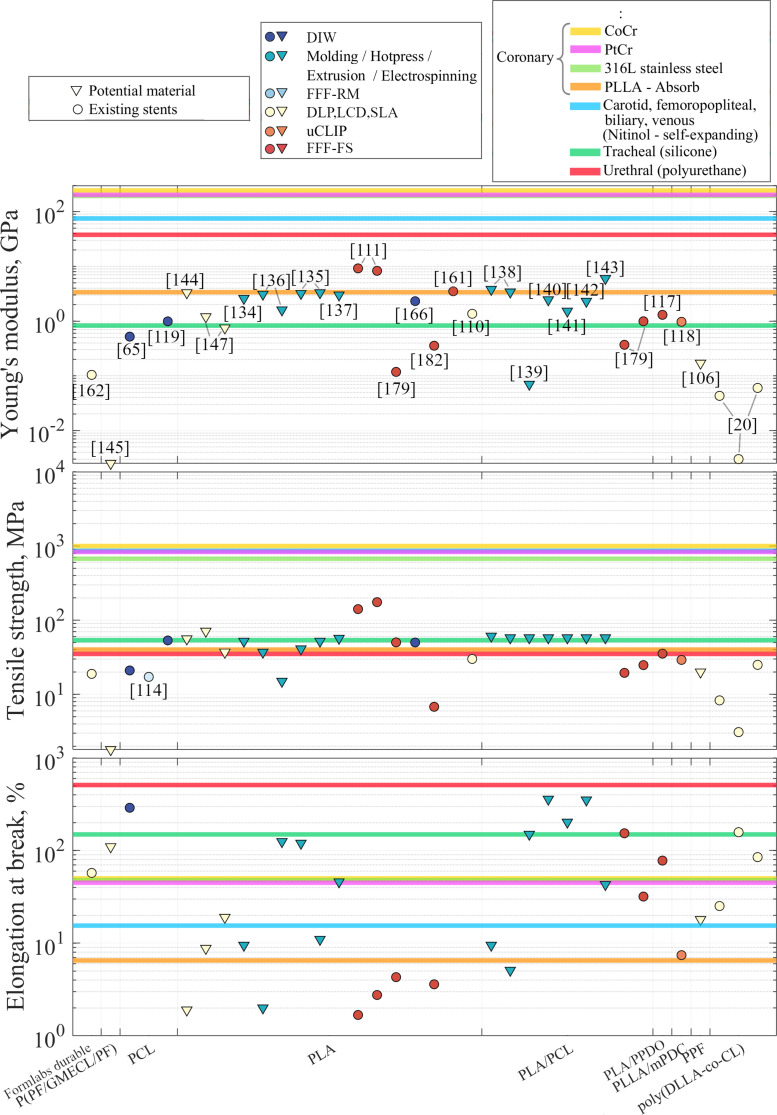
Material implementation in additive manufacturing of bioresorbable stents. The graph highlights the reviewed materials implemented in stents and materials that can potentially be used for stent manufacturing. One study is represented by one horizontal line and can have from 1 to 3 parameters (Young’s modulus, tensile strength, and elongation). Most bioresorbable stents have a Young’s modulus comparable only to silicone or PLLA material, not other clinically used metal stent materials. The tensile strength of BRS also remains lower than that of commercial stents. The elongation results even for the PLA materials have been higher than expected (up to 125%).

**Fig. 3.**
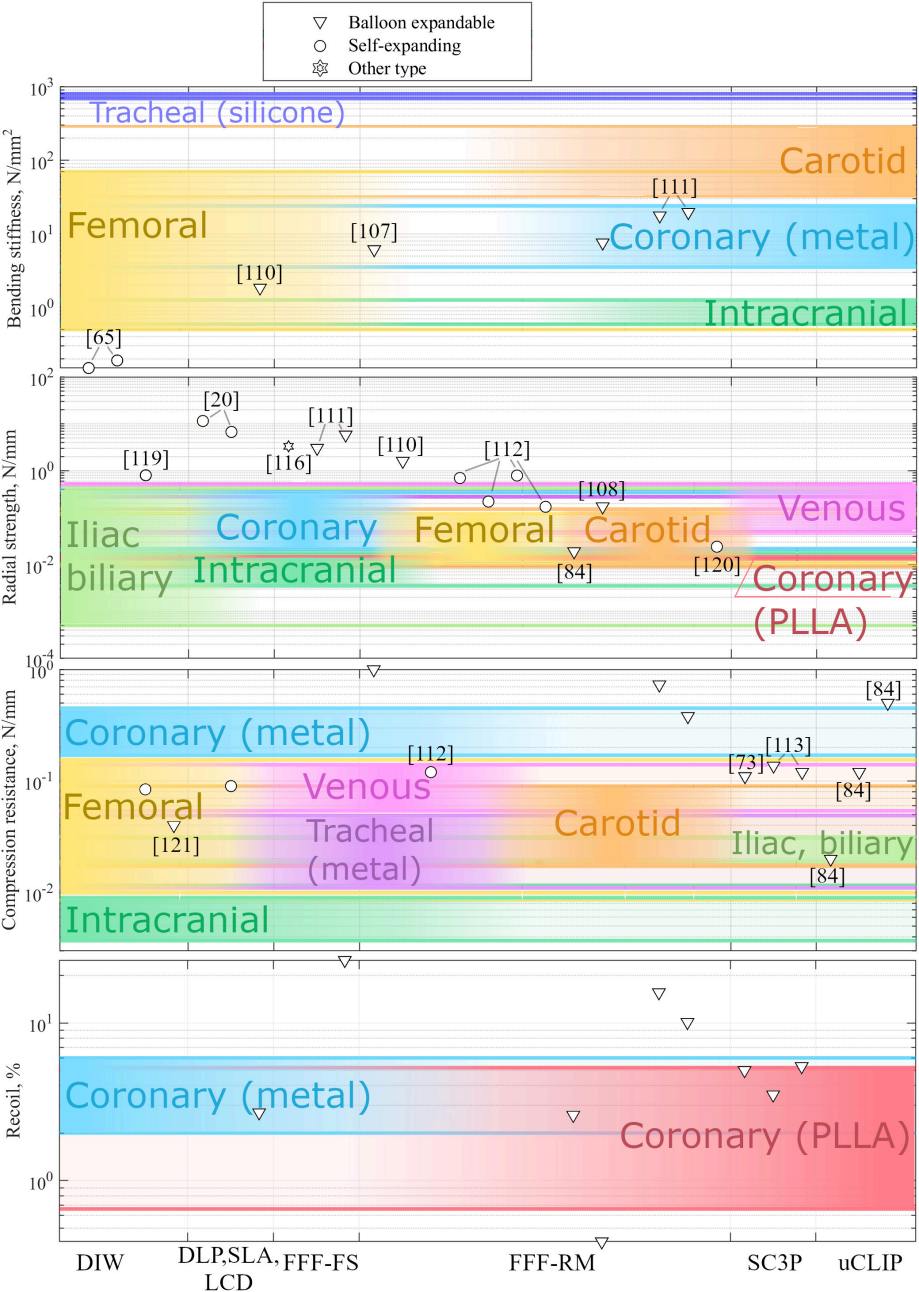
Mechanical parameters of bioresorbable stents and benchmark properties for various stent applications. One study is represented by one horizontal line and can have 1 to 4 parameters (bending stiffness, radial strength, compression resistance by parallel plate, and recoil). Although reducing bending stiffness is traditionally a major challenge in stent design to enhance flexibility, polymeric BRS may face challenges in achieving sufficient stiffness for applications such as carotid and tracheal stents. Radial strength is strongly dependent on stent wall thickness; higher results in BRS studies are usually achieved by scaling the thickness of the stent wall. Published results suggest that BRS can potentially replicate the compression properties of most commercially available stents, except those seen in intracranial applications. Several polymeric stents have been successfully developed with recoil performances comparable to those of coronary stents.

An analysis of AM BRS and bioresorbable materials under development is summarized in Figs. 2 and 3. Figure 2 presents materials that have already been used or have potential for implementation in the AM of BRS. Their main properties, such as Young’s modulus, tensile strength, and elongation at break, were mapped against the properties of clinically used stent materials for different stent applications. A comparison between these findings and the benchmark properties of currently used non-BRSs and BRSs fabricated by standard methods highlights the future directions of AM BRS development. We also provided the mechanical and geometrical properties of absorbable vascular stents to benchmark against non-AM BRS available on the market.

The common vascular coronary stent materials are cobalt chromium (CoCr) [73], platinum chromium (PtCr) [74], and 316L stainless steel [73]; PLLA [75] material was also taken as a benchmark coronary material because it was used in the most clinically advanced non-AM fabricated BRS: Absorb BVS. The most widely used self-expanding stent material, nickel titanium (Nitinol) [76], has also been analyzed. The other materials provided as benchmark are silicone [20], used for tracheal stents, and polyurethane [77], used for urethral stents.

In Fig. 3, the main stent mechanical parameters of AM BRS were also mapped against benchmark values of clinically approved non-AM non-BRSs for each application: coronary metal [78–82] and PLLA [80,83], carotid [84–86], femoral [87–89], intracranial [90,91], tracheal (metal [92,93] and silicone [94] material), venous [95], and iliac [96]. The same mechanical properties are chosen for all stents for comparison purposes. Radial strength results group properties of both self-expanding (chronic outward force) and balloon expandable (radial strength) stents. Similarly, compression resistance results group balloon expandable properties (crush resistance) and self-expanding (compression resistance).

**Fig. 4.**
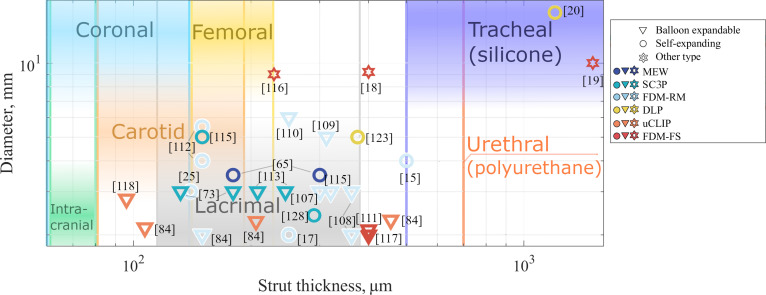
Diameter and strut size parameters of existing bioresorbable stents and benchmark properties for different stent applications. Thinner struts are associated with reduced incidence of early stent-related adverse events. From a geometrical perspective, only the thinnest intracranial application cannot be produced as an AM BRS.

The benchmark values for each application were considered the lowest and highest values reported in the literature. Perpendicularly applied load (parallel plate test) presents all clinically available and experimental data for all stent types, even though, according to ISO 25539-2 [97], compression and crush resistance to perpendicular load for self-expanding and balloon-expandable stents can only be applied to venous and non-aortic, non-corona, and non-renal arterial implant locations. Diameter and strut size of AM BRS (Fig. 4) in comparison with clinically used stents (coronal [98], intracranial [90], carotid [89,99], lacrimal [100], femoral [101,102], tracheal [103,104], and urethral [105]) can also provide information about the potential implementation of these devices in various applications.

### Analysis of the correspondence of mechanical parameters of clinically used stents and AM BRS

The analysis performed in Figs. 2 to 4 provides an outline of the capabilities and performance characteristics of AM methods and materials in BRS fabrication, toward the future clinical use of AM BRSs.

In Fig. 2, PLLA-based polymers and those containing additional additives are grouped under the general polylactic acid (PLA) category. PLA blended with PCL was placed in a separate category, PLA/PCL, as it is the most studied combination. It should be noted that most mechanical data for potential stent materials are obtained after molding, hot pressing, or extrusion. Because the final mechanical properties are substantially influenced by the manufacturing method, these results should be interpreted with caution.

Most BRSs have a Young’s modulus comparable to that of silicone (likely for self-expanding stents) and PLLA (most commonly in balloon-expanded stents). To date, many stereolithography (SLA)-fabricated stents have exhibited relatively low Young’s moduli. Fused filament fabrication printing on flat surface (FFF-FS) stents exhibit a wide range of mechanical properties; however, owing to their generally low precision, they are unlikely to be applied broadly. The Young’s modulus of PLA, the most extensively studied polymer for stent fabrication, can vary up to 9.2 GPa, highlighting its potential for tunable mechanical properties. PLA is commonly considered to have low elongation at break; however, some studies [106,107] have reported substantially higher values, up to 125% for certain blends. To further improve elongation, PLA/PCL blends are frequently used, achieving elongation up to 352% [108].

Owing to the wide variability in elongation, nearly all commercial materials can be theoretically mimicked using bioresorbable polymers. However, this fact alone does not support the feasibility of replacing commercial stents with polymeric ones unless their elastic and strength properties are adequately matched. The elongation at break is difficult to estimate because some sources do not explicitly state whether the elongation was measured at the break or at the maximum stress. However, the parameters were extracted where possible. The tensile strength of most polymeric stents is similar to that of polyurethane, PLLA, and silicone, but remains lower than that of commercial stents, as does their Young’s modulus.

When comparing stent parameters (bending stiffness, radial strength, compression resistance, and recoil) (Fig. 3), it is perhaps surprising that there has been no large gap between the reported properties of AM BRS and clinically used non-AM non-BRSs. Perhaps, the consistency at which these properties are achieved will remain the biggest hurdle to creating BRS AM stents. Most polymer BRSs in development offer potential for use in both coronary and peripheral applications. The most flexible stents in clinical use are femoral and intracranial stents, owing to the high degree of vessel bending in the femoral region and soft fragile intracranial vessels, respectively. Lower limb arteries present the highest levels of morbidity, and coupled with small bending radii [109], these stents tend to be flexible and self-expanding in nature. Coronary stents have slightly higher bending stiffness. Carotid stents share similar designs with femoral stents; however, their bending stiffness tends to be even higher than that of coronary stents. Although reducing bending stiffness is traditionally a major challenge in stent design to enhance flexibility, polymeric BRSs may also face challenges in achieving sufficient stiffness for applications such as carotid or tracheal stents. It is worth noting the extremely low bending stiffness value reported in Ref. [65]—0.15 N/mm^2^—achieved due to the manufacturing method used, namely, melt electrowriting (MEW).

Benchmarking data on radial strength indicate that polymer BRS can, in some cases, exhibit greater resistance to radial compression than clinically approved stents. While this may be beneficial in maintaining vessel patency, these results must be interpreted with caution. Radial strength is strongly dependent on stent wall thickness. Excessive thickness negatively affects vascular hemodynamics and renders the stents unsuitable for small vessels. For example, the high values reported in Refs. [20,110] are attributed to increased wall thickness, and the results in Ref. [111] are influenced by the unique sliding-lock design of the stent. In general, the performance of polymeric stents falls within the range of commercial stent benchmarks, suggesting their potential applicability in various clinical contexts. However, only a few stents [84,112] show sufficiently low radial strength, indicating the challenge of engineering low-stiffness polymeric stents that are also resistant to crack formation.

In terms of compression resistance, bioresorbable polymeric stents show a substantially broader range of values. Published results suggest that they can potentially replace most commercial stents. Some outliers [113–115] exhibit extremely high compression resistance, likely due to excessive wall thickness. It should also be noted that no intracranial clinical stents have been reported with such low compression resistance values.

The recoil parameter is highly dependent on stent design. Nevertheless, several polymeric stents have been successfully developed with recoil performances comparable to those of coronary stents. As expected, some stents exhibited recoil values that were too high for clinical applicability [113]. For strut thickness (Fig. 4), the documented thickness of self-expanding and balloon-expandable AM BRS was compared with their clinically used analogs. Thinner strut thickness is associated with reduced incidence of early stent-related adverse events, as demonstrated in multiple clinical studies [116].

The thickness data on the AM BRS are also encouraging in the sense that there are stents currently implemented in clinical practice with the thicknesses that AM technology can attain. Thin size BRS has not yet fabricated with high accuracy and repeatability using AM. There are several matches for coronal range with the thickness of 3D printed balloon expandable stents, mainly for the stent technologies producing lowest thickness struts—SC3P (solvent-cast 3D printing) [72] and μCLIP (continuous liquid interface production at the micro-level) [117]. There is more coincidence with the femoral implementation; they can also be produced by FFF printing on rotating mandrel (FFF-RM) [25,115], MEW [64], and SC3P [118] methods.

In terms of dimensions, stents for the carotid clinical implementation are slightly thinner than femoral stents. There are also many examples when this thickness has been reproduced in AM BRS by μCLIP [117], SC3P [72], FFF-RM [25,115], and MEW [64]. Silicone lacrimal stents have a large range of thicknesses and are larger than most metal stents. They can be fabricated using almost all of the 3D printing techniques. Tracheal silicone, similar to urethral stents, is even larger and has fewer problems in their 3D printing. Urethral stents have substantially lower reported data. In general, this graph allows the visualization of AM BRS stenting because if a technology requires a thicker strut or wall thickness, it can be produced by the method in question.

## AM of BRS: Process and Material Development

### AM technologies

#### Material extrusion-based FFF

##### FFF printing on flat surface

**Fig. 5.**
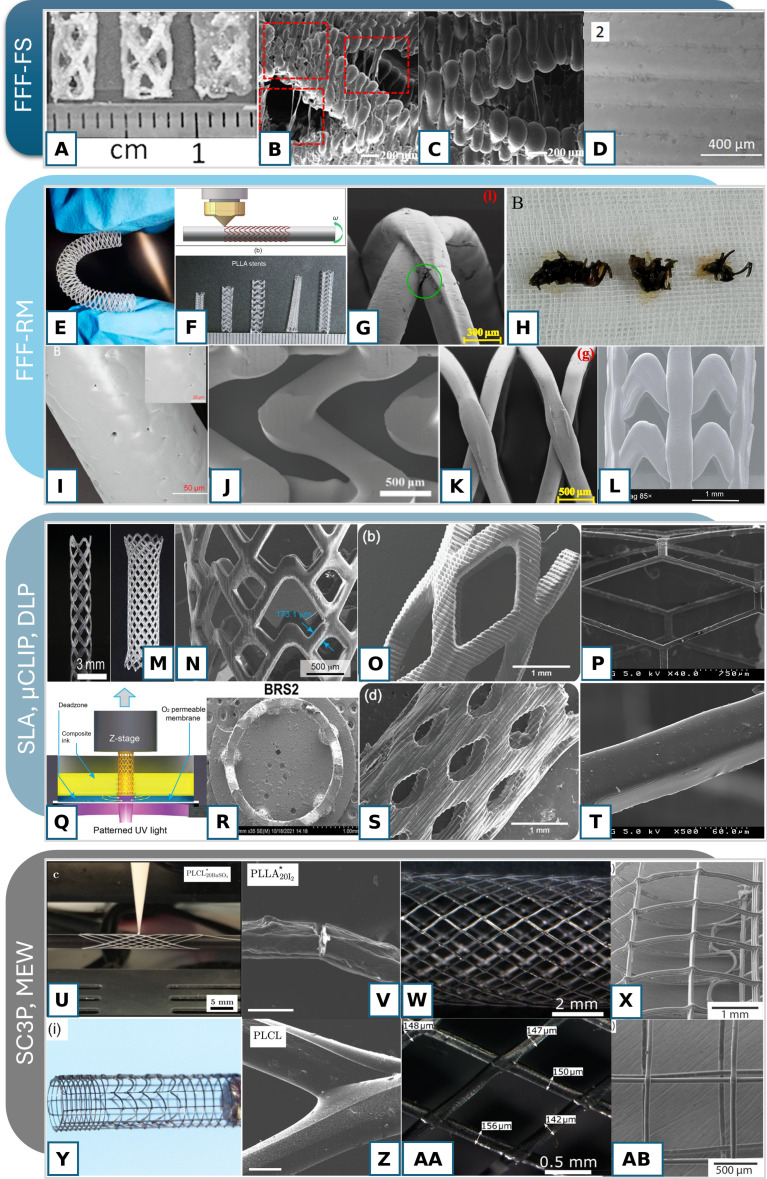
FFF-FS: manufactured stents have rough surface (A). The disadvantages of the method include overhanging strings of material in case of suboptimal printing parameters (B) [119]; visible filaments and potential voids between them (C) [119]; and rough surface of some materials can be smoothed with an appropriate coating: e.g., PLA (D) [18]; FFF-RM: stent for neurovascular application from PPDO (E) [25], scheme of the fabrication method for taper PLLA stents (F) [110], SEM of stent mechanical fractures (G) [120], stent fractures induced by in vivo degradation (H) [15], surface of the fabricated stent (I and J) [25,120], unfractured heat-treated PLA stent (K) [121], and PCL vascular stent (L) [111]. SLA, μCLIP, and DLP: stent overview (M) [115], surface quality and resolution of stents produced by Vat polymerization techniques: high surface quality of PLLA/mPDC stent (N) [110], self-expandable mPDC stent fabricated by DLP with pixelized surface (O), high quality of stents printed by SLA tubular method with collimator installation (P) [114] and scheme of the fabrication method (Q) [114], mPDC stent fabricated by μCLIP (R) [117], influence of higher shear stress on the surface (S) [122], and SEM of single strut (T); SC3P and MEW: scheme of the fabrication method (U) [123], surface morphology and crack of the iodine-containing stent caused by high brittleness (V), melt electrowriting of stents showing fiber deposition with layered (X), overview of the stent (Y) [65], and smooth surface of poly(l-lactic-co-ε-caprolactone) stent (Z) produced by the SC3P method [123]. High quality of printing of the solvent-cast PLLA stent (W and AA) [118], and single fiber structure (AB) [64].

The main limitation of FFF-FS is the surface quality, resolution, and the obvious post-processing challenges of forming tubular structures. The printed stents may have overhanging structures, visible filaments, or, in case of simple tubular stents, higher surface roughness than other printing methods (Fig. 5A to D).

The tested materials usually include PLA, which is a frequently used material for FFF and has good elastic and strength parameters but low elongation at break. The addition of PPDO with inverse properties resulted in the upgrade of elongation to 78% without substantially lowering other parameters [109]. Unfortunately, the deployment of this stent has only been characterized in FEA while the PLA and PLA/carbon quantum dot stents have been preconditioned at 37 °C and expanded to the maximum extent possible without failure [119]. The FFF-FS printed stents can attain 2 mm in diameter and 400 μm in thickness [119], which is insufficient for small vascular stents but is acceptable for other applications.

##### FFF printing on rotating mandrel

The limitations of the FFF for tubular structures, such as support for overhang features and poor surface quality, can be rectified by swapping the horizontal bed to a rotating mandrel. This technology does not require slicing and formation of material layers because of the generation of an uninterrupted toolpath for material deposition. The printed stents have a much smoother surface, but irregularities of the strut thickness were often present (Fig. 5), which can be further smoothed by post-processing [120]. The method allows for printing thinner struts (140 to 300 μm). The fracture morphology is a crucial parameter for BRS.

FFF-RM can be used for small-diameter stents starting from 2 mm [17] and is effective for both balloon [110,111,114,120–123] and self-expanding [15,17,25,115,124] stents. FFF-RM has been implemented for peripheral vascular [121], salivary gland [17], and biliary stents [15] because of its ability to print highly flexible structures. This capacity was demonstrated by Zhao et al. [115], where the authors evaluated the fatigue life of stents during cyclic flexion simultaneously monitoring the degradation and breakage of the struts. Moreover, drug coating was incorporated in PLLA stents printed using FFF-RM for coronary artery applications [125]. In other recent innovations, Khatami et al. [126] used a combined computational and experimental approach to fabricate PLA stents with variable thickness. To overcome the seam problem in FFF-RM, the biggest limitation of this printing form, Fang [112] used ultrasonic vibrations to join the seam to obtain better radial properties for stent applications.

#### Vat photopolymerization-based SLA and digital light processing

In the photopolymerization process, a photopolymer, which is a light-curable resin, is stored in a vat and treated with visible or ultraviolet (UV) light. The vat photopolymerization process is classified according to the curing method. SLA uses UV lasers to selectively cure a polymer resin and provides a smoother surface than FFF methods and a printing speed in the range of 10 to 20 mm/h. In the digital light processing (DLP) process, an entire layer of a 3D model is projected all at once, curing each point simultaneously, which can speed up the manufacturing process compared to SLA; however, each layer appears pixelized, and the accuracy of the printed part depends greatly on the projector resolution (Fig. 5). Continuous digital light processing (CDLP)/continuous liquid interface production (CLIP) and its modification, μCLIP, employ digital projection with light-emitting diodes and an oxygen-permeable window, which allows the liquid resin to flow between the interface of the printed part and the window. This increases the resolution, and the continuous movement of the build platform creates a smooth surface at speeds of several hundred millimeters per hour [127].

##### μCLIP

The development of μCLIP has improved the fabrication time, highlighting a new pathway for stent fabrication. The first articles on μCLIP reported the rapid fabrication of a 20-mm-long stent with a strut size of 150 μm in 10 min [113] and a 2-cm-long stent at 10 μm of layer slice thickness in 11.3 min [128] with a comparable strength to nitinol stents [129]. Further development of the last group demonstrated that μCLIP can fabricate BRS with 80-μm strut thickness using either support structures or sufficiently tall resin volumes [130].

The most frequently implemented material for μCLIP is mPDC. The latest advances in this material for stent μCLIP include reinforcement by micro/nanophase PLLA to increase mechanical properties, resulting in a Young’s modulus close to 1 GPa and a tensile strength of 29 MPa [130]. However, the elongation at break decreased to 7.4%, indicating the need for further material optimization. Mixing mPDC with iodixanol also slightly improved Young’s modulus (400 MPa) and decreased elongation [117]. This also rendered the stent radiopaque. However, the trade-off between radiopacity and mechanical properties must be considered. Efficient visualization with 5 wt% iodixanol is possible for the strut thickness equal to 456 μm while increasing iodixanol leads to more strut fractures during the crimping and expansion processes. However, an SLA tubular method (a mixture of SLA and FFF-RM ideas) was recently introduced [131]. The authors proposed a rotating SLA printer for stents with struts as thin as 70 μm with high accuracy, speed, and stability.

##### Digital light processing

The mPDC has also been implemented in DLP stent printing [116]. However, it showed low mechanical properties (Young’s modulus of 13 MPa in compression) and a pixelized surface. An alternative application of the soft material have been proposed by Paunović et al. [20]. The tracheal stent with high thickness (1.2 mm) and patient-specific geometry demonstrated equivalent mechanical properties to existing silicone stents, good in vivo results, and a low rate of migration. The DLP material proposed for this purpose was poly(D,L-lactide-co-ε-caprolactone), which was combined from 2 types of polymers with optimized ratios.

#### Direct ink writing

Direct ink writing (DIW) consists of 3D printing by direct extrusion of material without any external heating using pneumatic, motor, and screw pressure. Layer-by-layer fusion can be performed by direct material joining with or without the help of external sources, such as UV light, a heater, or another energy source. DIW can print composite materials and drug-loaded polymers for stent applications. However, it is difficult to control the process and to achieve high geometric dimensions and surface roughness accuracy.

##### Solvent-cast 3D printing

During SC3P, polymers are dissolved in a volatile solvent that evaporates during deposition to produce a solid polymer filament. The material lately reviewed for SC3P include PCL and PLLA. Singh et al. [132,133] explored the fabrication of PCL and polycaprolactone-carbon iron particle (PCL-CIP) composite stents and Schieber et al. [118] evaluated the use of PLLA for SC3P. The optical imaging of PLLA stent verified a highly regular structure with junctions properly welded, leading to a flexible and resistant prototype (Fig. 5).

The choice of an appropriate radiopaque material showed good results for PLLA/10 wt% iodine and PLLA/10 wt% BaSO_4_ in terms of strut thickness (208 to 245 μm) and compression strength [134]. Iodine resulted in increased brittleness that can cause stent fractures and BaSO_4_ rendered a rough surface that can be disadvantageous for stents if the roughness is too high (Fig. 5). The last developments in DIW stent manufacturing included functionalized material surfaces with increased cell adhesion and hemocompatibility [135]. The strut thickness of SC3P stents can achieve 130 μm, which is in the range of strut thickness for CE-marked stents or released to clinical trials BRS (Absorb, DESolve, Fortitude, Acute, Magmaris, Xinsorb, Art Pure, Ideal, and ReZolve), which was commonly between 120 and 150 μm [136]. However, in case of material additives and coating to increase mechanical properties, this value reaches 180 to 240 μm [72,134].

##### Melt electrowriting

MEW is a 3D printing technology that uses high voltage to charge and stretch the extruded fiber during deposition. Printing polymer melts with high viscosity and low conductivity allows for the deposition of fiber networks with diameters of 5 μm that can be deposited in a layer-by-layer manner to millimeter heights in a direct-writing manner [64]. The material chosen for the assessment of stent printing possibility was a nanocomposite of PCL and reduced graphene oxide (PCL-rGO) for augmentation of mechanical properties. The resulting Young’s modulus reached 510 MPa with a tensile strength of 21 MPa, which is sufficient for certain stent applications. Surface analysis proved the possibility of producing highly repeatable struts with a thickness of 60 μm. However, the challenge of MEW-based stent device fabrication is that the increase in strut thickness and width is only possible by layered fabrication, creating visible fibers and a rougher surface. Struts thicker than 60 μm may be required to increase the radial strength in polymer stents; however, the layered strut smoothness and structural integrity under radial load and degradation should be studied.

#### Selective laser sintering

Powder bed fusion-based selective laser sintering (SLS) produces the required geometry by laser scanning a powder layer [137]. It is the least explored method to produce BRS for the current moment [137]. Flege et al. [138] reported the fabrication of PLLA and PCL stents using the SLS process. However, the fabricated stents exhibited 2% porosity, which resulted in poor surface roughness, which was reduced using the spray coating method.

### Bioresorbable material developments

#### FFF materials

Until now, PLLA has become the most widely used substrate for printing BRSs [139]. As a result, FFF is an accessible option for producing stents since 3D printing from PLA family filament is a well-established process. The existing properties of PLLA filament are usually in the range of 2 to 4 GPa [136]; however, the increase of Young’s modulus to 7 GPa, tensile strength to 450 MPa, and elongation at break to 23% has been reported for PLLA filaments fabricated by a specific technique of melt-spinning and hot-drawing [140].

Moreover, the bioresorbable material development is rapidly evolving and new materials can be explored for AM fabrication of stents. Some PLLA nanocomposite materials have been proposed as suitable for BRS. The PLLA with 15 wt% stearic acid-modified BaSO_4_ nanofillers showed great Young’s modulus (6 GPa) but lower elongation at break (35%) [67], while 15 wt% L-lactide functionalized tantalum dioxide (Ta_2_O_5_) and 10 wt% L-lactide functionalized hydroxyapatite (HA) presented lower but still acceptable Young’s modulus (around 3 GPa) and much higher elongation (70% to 90%) [141]. The mechanical properties were also improved in the modification of PLLA with triacetin, Pluronic F127, and magnesium hydroxide nanoparticles [142]. The mechanical properties reached 2.6 GPa in Young’s modulus and 52 MPa in strength. The elongation was only 9.5%, although elongation of pure PLLA in their tests only equaled to 4.3%, which suggests that for another type of PLLA pretreatment, the elongation of the resulting material can be increased. The drawback of the article is the lack of mechanical characterization after the addition of nanoparticles, which can influence the parameters.

The investigation of PLA has also given some promising results. The addition of poly(ethylene glycol) (PEG) indicated an optimum in mechanical properties for 5 to 10 wt% of PEG because 5 wt% gives good elongation (120%) while 10 wt% lowers the elongation (11%) but increases tensile strength to 52 MPa [143]. The addition of poly(ethylene oxide) (PEO) to PLA has shown great results in terms of elongation (139%) although some refinement of PEO ratio or molecular weight is also needed to approach Young’s modulus (1.6 GPa) and strength (15 MPa) to neat PLA [144]. PLA blend with 10 wt% poly(butylene succinate-co-adipate) also provided adequate mechanical properties close to PLA but with improved elongation (45.8%) [145].

PLA/PCL blends may offer great potential as BRS material as the PCL can improve the elongation at break. However, other properties are also substantially affected by the ratio of PCL. The change of PLLA:PCL ratio from 95:5 in extruded material [106] to 50:50 in electrospun fibers [146] resulted in the decrease of Young’s modulus and tensile strength from 3.4 GPa to 70 MPa and 57 to 4 MPa and an increase of elongation by 145%. Different additives can give a positive effect on the mechanical properties of this blend. The addition of 5 wt% of Mg–Zn–Y microparticles improves the elongation by 4% and gives radioopacity [106]. The blend of PLA/PCL with 10 wt% cellulose acetate butyrate increased the elongation to 359% without substantially altering other properties [107], while the blend with 2 wt% of polyoxymethylene increased the elongation to 203% but also slightly improved the tensile strength [147]. Another possible variation is the blend of PLLA and 30 wt% diblock copolymer 5-armed PCL-block-PLLA that increased the elongation to 352% and only slightly decreased the Young’s modulus and strength compared to pure PLLA [148]. The triblock copolymers PLLA-P(LLA-co-CL)-PLLA have shown even more potential with 6 GPa Young’s modulus, 70 MPa strength, and 43% elongation [149].

#### SLA and DLP materials

The main disadvantage of currently existing bioresorbable resins for SLA and DLP is the low mechanical properties of resulting materials. As well as for the FFF-based fabrication methods, for the PLA-based resin, the elongation is the most crucial limitation. The 2-armed poly(D,L-lactide) macromer with a molecular weight of 0.6 kg/mol per arm and Irgacure 2959 as photoinitiator showed only 1.9% of elongation while showing good Young’s modulus (3.3 GPa) and tensile strength (56 MPa) [150]. However, it is important to note the positive trend in flexural characteristics with the increase of number of arms. In contrast, a CLIP DLP-printed ABA triblock poly(propylene fumarate-b-γ-methyl-ε-caprolactone-b-propylene fumarate) copolymer with the ratio 5:16:5 showed exceptional strain at failure at 37 °C (110%) but insufficient Young’s modulus (2.5 MPa) and tensile strength (1.8 MPa) [108].

Poly(propylene fumarate) (PPF)-based resins are a well-known bioresorbable SLA- and DLP-compatible material. Depending on the formulation, this photopolymer usually has low Young’s modulus (130 to 170 MPa [151]). However, there is evidence that it can be tuned in a range of orders of magnitude depending on laser pulse fluence during SLA [152]. Also, the addition of HA nanoparticles to a molded PPF has shown to increase the tensile, flexural, and compression modulus to 1 to 2.1 GPa, which can possibly be translated to SLA- or DLP-printing methods [153].

A recently proposed DLP material—poly(L-lactide-co-ε- caprolactone-co-N-vinyl-2-pyrrolidone) network—could be a promising candidate for stent 3D printing [154]. The formulation containing 50 wt% fumaric acid monoethyl ester (FAME) functionalized 3-armed poly(L-lactide) prepolymer (3-PLLA-F) with 50 wt% N-vinyl-2-pyrrolidone (NVP) showed medium Young’s modulus (1.2 GPa) and good tensile strength (71 MPa) but had low improvement of maximum elongation (8.8%) compared to PLLA. However, replacement of 15% of 3-PLLA-F with poly(ε-caprolactone) prepolymer (2-PCL-F) decreased the Young’s modulus and tensile strength to 0.75 GPa and 37 MPa, respectively, but resulted in a substantial increase of elongation to 19%. Therefore, a lower amount of 2-PCL-F could potentially give optimal properties for small-diameter stents that do not require high elongation. Another potentially interesting SLA material was proposed in Ref. [155]. Although the material has only been characterized in compression with the strength in the range of 470 to 540 MPa, this corresponds to 57% to 66% of PLA compressive strength.

## Future road map for stent AM

### Future directions for stent 3D printing

**Fig. 6.**
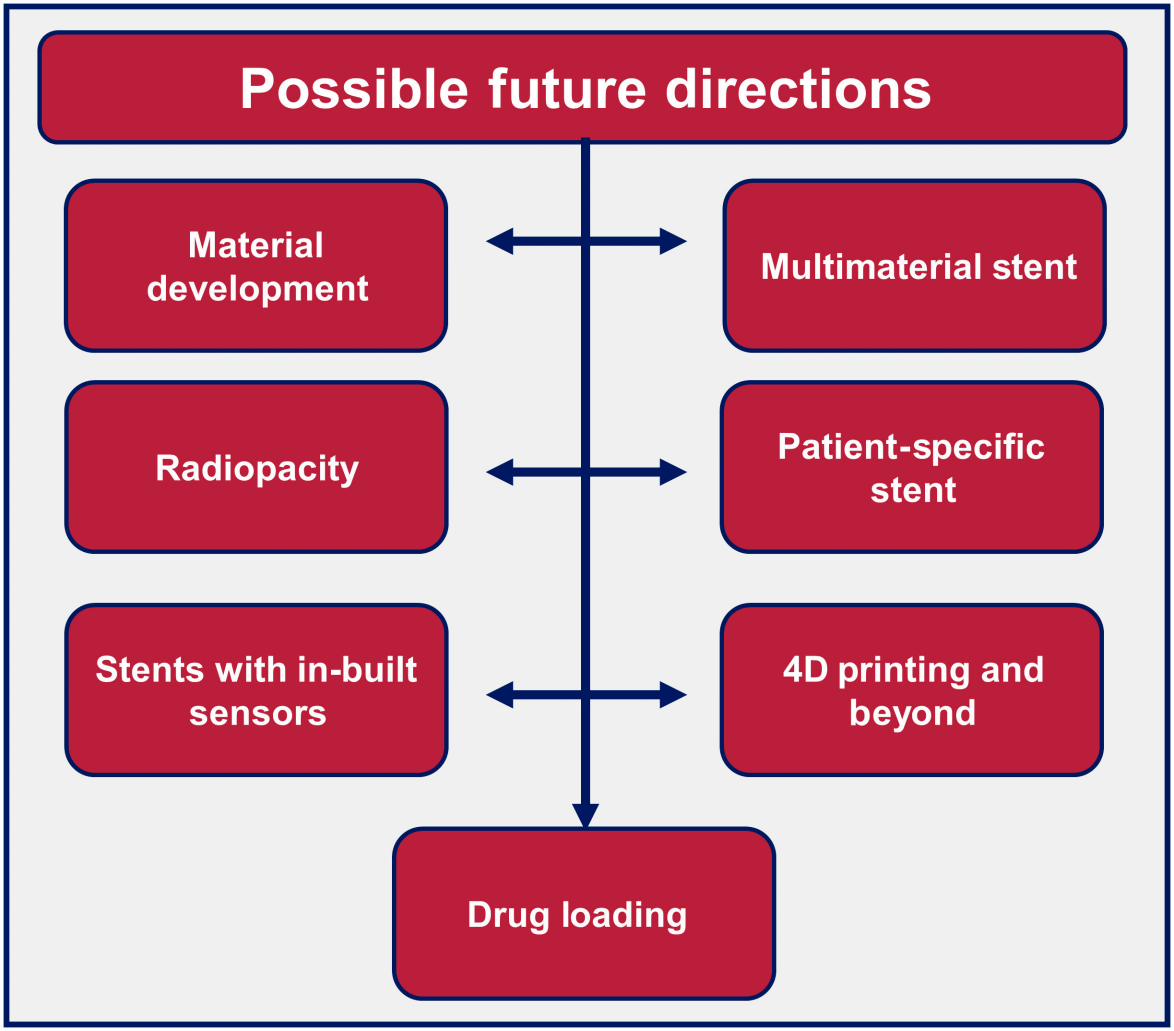
Possible future directions and milestones for AM-based BRS.

AM methods in cardiovascular stent production can be classified as a single-step process that replaces microtube production through extrusion, tube drawing, and subsequent laser microcutting. Although AM is an emerging manufacturing technique, its efficacy of AM is expected to outperform that of traditional stent manufacturing methods. Despite this, only a limited number of studies have been performed to fabricate stents. The use of ultrathin struts with a thickness of less than 70 μm [156] is the most challenging feature for the present AM industry [56,157]. Possible future directions and milestones from the literature review are presented in Fig. 6 and discussed below.

Powder-based AM technology has the advantage of fabricating different biodegradable materials such as iron [157], zinc [56], magnesium families, and other polymer materials PLA, PCL, and PLGA. However, a detailed study of the process parameters is required to achieve certain dimensions with SLM systems. The small size geometry requires special attention to the process parameters during printing. Finazzi et al. [158] presented complete design rules for the AM of stents without SLM support. In traditional SLM systems, the scale of the powder feedstock, laser beam location, and sheet size are equivalent to the strut size. The standard powder size range for SLM systems must be between 15 and 45 μm, with beam sizes varying from 50 to 100 μm and sheet thicknesses ranging from 25 to 100 μm. Although these measurements are in substantial for broad materials, they determine the method resolution for thin stent struts. However, cracks and other defects remain a challenge during stent expansion. In addition, the surface of SLM-printed stents is too rough, which always requires post-processing treatment, such as electropolishing. The post-processing techniques improved the surface roughness and decreased the strut thickness, which directly affected the mechanical strength. Subsequent heat treatment and surface finishing activities should be studied further to thoroughly study productivity. In addition, only the zigzag shape has been explored for the SLM to avoid support during printing. FFF as a method of BRS fabrication has advantages over other 3D printing methods. First, the temperature of production in FFF is comparatively low. Therefore, the common issues of SLM for low-temperature materials, both material-related harm and low surface roughness due to the higher processing temperature, may be effectively avoided in FFF. In comparison with another widely used method, SLA, where parts can only be produced from photocrosslinkable materials, the range of building materials for FFF is far more diverse. FFF-RM is the most suitable AM method for producing self-expanding structures that mimic braided stents. Braided stents are considered the standard for gastrointestinal self-expanding stents [159] and give better results in peripheral applications [26]. Therefore, FFF allows stent fabrication with good flexibility and conformability and has greater potential for gastrointestinal and peripheral vascular stents, as well as neurovascular applications [25]. Conventional FFF-FS stent production is associated with some difficulties. The stents have complex networked spatial architectures consisting of various overhanging parts. Therefore, large support systems inside and outside the stents must, at the same time, be printed simultaneously during standard FFF-FS to carry the stored overhang parts, causing post-treatment complications and reducing manufacturing performance. Furthermore, separation from these support systems can damage or deform some intravascular stents. Finally, post-treatment implementation may limit the dimensions and structures of the engineered stents. In the future, where a patient’s vascular parameters would be measured using imaging tools, accompanied by customization of the stent configuration and on-the-spot printing of BRSs, rapid fabrication would be the most major requirement. The period needs to be brief enough that the whole procedure from parameter evaluation to stent distribution takes less than 20 min [160].

The results of some research papers presenting new polymer BRS must be interpreted with caution if the stent has been tested as printed in expanded or unexpanded form without performing all the required crimping and expanding steps and fracture analyses. For complete characterization, balloon-expandable stents should be crimped on a delivery system to highlight their behavior during delivery and after expansion from the delivery system for the real stent properties. Self-expanding stents should also have an additional step comprising stent crimping and expansion in the case of unexpected crack appearance. Phantom studies of stent deployment [117,134] can be helpful as a first step in deliverability evaluation, whereas in vivo studies can provide more detailed information.

### Multimaterial stent

Multiple printheads may be used simultaneously during printing, allowing the 3D printing of heterogeneous polymeric stents with a variety of polymers and on-demand adjustment of mechanical and biological properties. In addition to fabricating polymeric stents, 3D printing technology can be used to fabricate other multilayered tubular constructs, such as 3-layered artificial blood vessels in tissue engineering [161] and double-layered hollow fiber membranes for gas separation [162]. For the extrusion process, multimaterial printing is much easier than other AM methods. An example is printing a few layers of PLA and further printing of PCL on PLA or vice versa [163]. However, with advanced pneumatic-based extrusion system printing, multimaterial printing is not limited to polymers. It can be used to print polymers with other materials, such as hydrogels and metal particles. The example of printing PCL with grooves in the stent design and later printing hydrogel in the grooves for radiation esophagitis study is given in literature [164].

The printing of metals and polymers together combines the benefits of metal strength, polymer resorption, and drug delivery properties. Jang et al. [165] combined the braided nitinol and 3D printed PCL stents to have multiple advantages for treating unresectable malignant hilar obstruction. However, both metal and polymer materials can be degraded. DES stents also consist of polymer layers for drug-eluting features on metal stents that withstand the pressure of arteries. However, AM methods can provide freedom to design the stent geometry with metal and polymer materials for specific strength, resorption, and drug-eluting time.

### Stent radiopacity

Stent imaging methods for visualization during and after implantation can be invasive, such as fluoroscopy, CT, and MRI, or noninvasive, such as intravascular ultrasound (IVUS), optical coherence tomography (OCT), and angiography. Common polymer and metal BRS are fully radiolucent and can only be imaged with IVUS; therefore, noninvasive imaging is impossible.

As a result, stent observation necessitates an additional process, such as the incorporation of markers at the distal and proximal ends, which does not represent the stent framework and expansion [166] and can give inaccurate information due to marker dislodgement during scaffold deployment [67]. AM methods allow the inclusion of contrast agents during printing to improve the radiopacity. These agents can be in the form of liquid, nanoparticles, or microparticles and contain iodine [167], BaSO_4_ [67,134] or zinc and yttrium [106]. The effectiveness of radiopacity was demonstrated for PCL and PLLA in FFF, DIW, and laser cutting. Different contrast powders are available on the market, such as Gadolinium, Lomeron, Lomeron400, Lopamiro, Barium Sulfate, ProHance or SonoVue. However, the addition of radiopaque components can result in thicker struts.

### Patient-specific stents

Patient-specific implants are typically characterized by a unique shape dictated by the patient’s anatomy. The use of patient-specific information to design CAD models for 3D printing has already shown its effectiveness for orthopedic implants [168–170]. For stents, this geometrical fit, obtained by CT angiography and fluoroscopy, may effectively reduce stent restenosis by avoiding or substantially decreasing the interaction stress between the stent and the artery wall [171]. Another possible approach to patient-specific design is the adaptation of the strut thickness. They should be altered in a way that does not generate stress concentration at the stent ends and adapts to the vessel’s shape with thinner struts in unstressed areas to avoid endoluminal paving and thicker struts in strained areas to prevent strut breakage.

However, the most frequently used technology in recent years, FFF-RM, only allows for the printing of tubular structures. Vat technologies have also focused on classical designs. The existing vascular patient-specific printing approach involves the alternation of tubular segments with different designs to increase compliance. In tracheal stents, a recent article successfully printed a fully patient-specific stent based on rabbit anatomy and compared its performance to that of a commercial silicone stent. Further development of 3D-printing techniques for anatomical stents will require evaluation of their delivery and deployment. Stent printing time can become a limiting factor in case of patient-specific stents. The recent commentary on BRS for the treatment of airway collapse in children highlighted that there are successful outcomes using a 3D-printed BRS to treat difficult airway and BRS can be easily implanted but that technique has the disadvantage of long preparation times that are necessary to produce the stent [172]. Some techniques allow for fast (7 min, FFF-RM [17]) or both fast and precise printing (μCLIP [128]), although the speed parameter is not discussed in most reviewed articles. Auxetic structures [173] can further help achieve better shapes required for patient specificity.

Multibranched stents are another important possibility for AM. The feasibility of a bifurcated stent design was demonstrated using SLM in the literature [158], further expanding the fabrication of patient-specific stent designs. However, no studies on bifurcated polymer BRS have been identified.

### Stents with in-built sensors

AM provides the opportunity to print stents with in situ assembly of sensors layer-by-layer. For example, Park et al. [174] created a wireless pressure sensor integrated into a 3D-printed biocompatible and biodegradable PCL polymer stent. The patient will monitor the development of unexpected restenosis and manage the medication dose by measuring blood vessel pressure with an implanted smart stent. Furthermore, because it is constructed of silicone, the smart stent is not affected by magnetic fields from surgical instruments such as MRI. It also decomposes and is consumed by the body after surgery. The wireline pressure sensor exhibited a resonance frequency of 137 to 150 MHz. The results of the in vitro tube test demonstrated that the frequency of the wireless pressure sensor had high linearity within the 0 to 120 mmHg pressure range and a 50-kHz/mmHg sensitivity. Owing to its high flexibility, the manufactured wireless sensor can be attached closely to the inner walls of the stent and is fully absorbed over time.

In contrast, using built-in sensors, patient health data can be easily captured for analysis using the Internet of Things (IoT). The medical IoT is a powerful enabling technology for implantable medical devices, such as innovative stents. The IoT is a network of devices that can connect wirelessly and send data to cloud-based servers. In the context of the medical IoT, this may be a ward-based monitoring system. These “things” may be stored and communicated on the cloud to make patterns and forecasts, allowing for “big data”, huge quantities of particular and unspecified personal data. The effect of these gadgets on health care is becoming commonplace, contributing to the decentralized health care paradigm [175]. The stent has also been studied theoretically and in vitro as an antenna for wireless power transfers. The relevance of wirelessly charging devices stems from the dangers of implanting chemical batteries within the body and the size restrictions imposed by stents [176]. Active devices, such as those powered by a battery, are vulnerable to leakage, corrosion, and excessive heat generation [177]. This limitation can be rectified by AM methods that allow the development of new materials, such as wireless power transmission.

By transferring power wirelessly to a stent, the battery can be eliminated, reducing these dangers. As the IoT expands, the value of AM with sensors will become more apparent for smart stents. Implantable biodegradable sensors have been found to be comparable to the materials used in stents. Hwang et al. [178] developed sensors based on PLA to create PLLA and PDLLA copolymers. In a series of studies, PLA derivatives with various construction phases using silicon electrodes and magnesium interconnects were shown to produce biodegradable semiconductors.

### 4D printing and beyond

Although 3D printing is often considered a highly versatile manufacturing method, certain limitations must be addressed. One possibility is that a 3D printed part with initial state parameters is assumed to be static [179]. This may be solved using a new 4D printing concept. It was introduced in 2013 and produced structures that can be preprogramed to attain a required shape under the expected conditions [180]. External stimuli can alter the shape and behavior of printed parts. Temperature, pressure, magnetic field, and pH are all variables that can act as stimuli. Hydrogels, dielectric elastomers, memory polymers, and liquid crystal elastomers are used to create exceptional 4D printing materials for different applications [181,182]. Because it is a new concept, only a limited amount of work has been performed for stents. Wei et al. [183] demonstrate the fabrication of stent using shape memory polymer by the DIW technique. PLA polymer with Fe_3_O_4_ was mixed with dichloromethane as the solvent and benzophenone as the photo-initiator. The material exhibited a shape memory effect with the help of an external magnetic field. After removing the shape memory stimulus, approximately 97.5% shape recovery was achieved. The spiral stent expansion was demonstrated by a physical setup by placing the stent in a tube and applying a magnetic field. The stent was fully expanded within 10 s and showed the candidacy for self-expandable stents. However, current shape memory materials with a wide range of capabilities (self-healing, dual activation mechanisms [184]) show low mechanical properties and need further developments to be successfully implemented as stents.

Additionally, 5-axis 3D printing, often known as 5-dimensional 3D printing, is a future advancement in 3D printing (5D printing). This was initiated by Mitsubishi Electric Research Laboratories in 2016. This method uses a movable print head that can move at 5 different angles of inclination. Instead of producing flat layers as in 3D printing, this technique can create curved structures all at once [185]. To explore shape memory stent using AM, parallel research and development in 3D, 4D, and 5D printing is needed.

### Drug loading

AM methods, especially for polymers, allow drugs to be loaded into the material for drug release with stent resorption [186]. AM processes can facilitate the incorporation of drugs during layer-by-layer assembly, thus facilitating release profiles that are not possible using current BRS fabrication methods. Nevertheless, high-temperature printing or UV curing can damage some therapeutics during printing. One solution is to print at low temperatures. Kim et al. [17] printed PCL at 70 °C with amoxicillin and cefotaxime drugs. The low-temperature printing maintains the properties of the drugs. However, in-depth exploration is required to check the effect of energy parameters such as extrusion temperature and UV curing on the performance of drugs. SC3DP is one of the viable solutions for the addition of drugs in the printing material, which will not damage the properties of the drugs. However, the choice of solvent to avoid any kind of reaction with the drugs is also important [187].

The medication should be released in a regulated manner; therefore, the stent breakdown period should be tailored. Furthermore, utilizing this approach to produce stents helps one introduce medications and other materials with additional properties that can add value to the cardiovascular stent, thus enhancing its efficiency. Furthermore, 3D printing has the potential to be used for ureteral stents [188] with drug loading.

### Regulatory challenges

Despite the technological promise of AM for BRS, regulatory pathways remain a substantial barrier to their clinical adoption. Unlike traditional stent manufacturing processes, AM introduces additional variables, including material processing, design customization, and mechanical performance, that challenge current medical device regulations. Regulatory bodies such as the FDA (U.S. Food and Drug Administration) and EMA (European Medicines Agency) require comprehensive validation of device safety, efficacy, and manufacturing traceability and reproducibility. However, many AM methods still lack the standardization necessary for consistent quality assurance across manufacturing batches, especially at the microscale resolution needed for vascular stents [189]. Uniquely, for endovascular devices, their delivery catheters typically assume that stent-based devices are symmetric and do not control their orientation, which is an additional consideration in their design and deployment.

Furthermore, bioresorbable materials introduce an additional layer of complexity due to their degradation behavior in vivo; however, the recent FDA approval of Abbott’s Esprit stent is a promising step forward in this regard. Manufacturers may need to create bespoke testing protocols to address both mechanical integrity and biodegradation timelines, which will prolong the regulatory approval process. 3D printed orthopedic medical devices, including customized devices, show a pathway for regulatory approval of patient-specific devices.

The absence of a harmonized framework for patient-specific devices—especially those fabricated point of care—further complicates regulatory compliance. Customized AM stents challenge traditional approval routes that are designed for off-the-shelf, mass-produced devices. Although the FDA has released guidance on technical considerations for AM devices, these documents remain general and are not tailored to BRS or cardiovascular applications specifically [190]. To advance AM BRS toward clinical use, collaboration between regulatory bodies, researchers, and industry stakeholders is essential to define risk-based, application-specific standards. These must encompass not only biocompatibility and performance but also manufacturing reproducibility and traceability.

## Summary

AM is an emerging technology in the biomedical sector with the ability to customize devices that address patient-specific clinical challenges. If the challenges of reliably printing with micrometer-level precision and at scale are overcome, AM can play a major role in the development and fabrication of the next generation of stents. It serves as a valuable research tool, offering flexibility to print a wide range of metals, polymers, or their composites. The capacity to fabricate materials with super-elasticity and embedded sensors could provide meaningful improvements in procedural outcomes.

Next-generation AM stents should consider the following challenges and opportunities:

• thin strut thickness and sufficient radial strength to tolerate vessel recoil;
• balancing stent resorption period with vessel remodeling or healing time;
• the placement technique must be suitable for implanting stents with minimal tissue trauma and delivery system customization;
• a uniform expansion profile, particularly where AM parts are produced from FFF-RM, where a seamline is induced;
• sufficient radiopacity to be visualized using conventional imaging techniques; and
• incorporating sensors to facilitate remote health monitoring.

Although the most recent vascular stent AM technology is still in the research and development stage, it has substantial promise, as reported here. The AM BRS is a valuable prototyping tool, irrespective of how quickly it translates into devices approved for clinical use. The implementation of AM BRS in clinical practice is complex, not least because BRS has yet to displace DES.

The mechanical performance of AM BRS is a crucial barrier to clinical adoption. Therefore, we identified the main material and stent parameters and compared the existing literature on AM BRS and stents commercially used in clinical practice. The commercial stents were divided into categories according to their implementation, which allowed us to highlight which AM technique can produce stents comparable to clinically used ones based on evaluated parameters.

We determined that FFF-RM can produce bending stiffness similar to that of some existing stent types; however, available data on AM BRS are scarce. The radial stiffness of some AM BRSs corresponds to that of coronary metal stents, and the COF of some self-expanding FFF-RM stents is close to that of clinically used femoral benchmark parameters. The same tendency was observed for balloon-expandable stents, with some properties similar to those of conventional coronary stents. Stent recoil of AM BRS also appears to compare favorably with existing stent performance. This indicates that polymer AM BRS has the potential to offer sufficient mechanical performance in a host of applications; however, this cannot be compromised by excessive strut thickness or variability in mechanical performance.

We also studied the material parameters of both AM BRS and materials deemed suitable candidates for them. The Young’s modulus and maximum tensile strength were lower for the polymer BRS and could only be compared to silicone stents and PLLA Absorb stents. All the metal materials for the stents had much higher material properties. However, in the case of elongation at break, the data were more distributed, and materials used for AM stents can have the same elongation at break as commercial stents if not better. Finally, we examined the diameter and strut thickness that current AM technology can produce in stent fabrication. Only for intracranial applications is there no option for polymer BRS implementation. All other applications can become areas in which polymer BRSs can compete, if consistent mechanical performance and dimensional requirements are met.

## Data Availability

The datasets used and/or analyzed during the current study are available from the corresponding authors on reasonable request.
