# Supplementary material for: Additive Manufacturing of Polymeric Bioresorbable Stents: A Mechanical Performance Perspective
Source: Biomater Res. 2026 Jan 23;30:0259. doi: 10.34133/bmr.0259 (PMC12827891; doi:10.34133/bmr.0259)
Supplement: Supplementary 1 — Supplementary Methods [file bmr.0259.f1.docx]

Supplementary Materials for

**Additive Manufacturing of Polymeric Bioresorbable Stents: Current Progress and Future Directions**

Gurminder Singh^+1,2^, Irina Khaydukova^+1^, Kevin Walsh^3^, Colin J. McMahon^3,4^, William Ronan^5^, Eoin D. O’Cearbhaill^1^

^1^School of Mechanical and Materials Engineering, UCD Centre for Biomedical Engineering, University College Dublin, Dublin, Ireland

^2^Department of Mechanical Engineering, Indian Institute of Technology Bombay, Mumbai, India

^3^Department Paediatric Cardiology, Children’s Health Ireland at Crumlin,
^4^School of Medicine, University College Dublin
^5^Biomedical Engineering, University of Galway, Galway, Ireland.

^+^ denotes equal contribution

^*^Corresponding Author: [eoin.ocearbhaill@ucd.ie](mailto:eoin.ocearbhaill@ucd.ie), gurmindersingh2012@gmail.com

The PDF file includes:

- Supplementary Methods

**Supplementary Methods**

**Search strategy**

The search was conducted through Scopus database using the search query TITLE-ABS-KEY(stent AND (finite OR element OR modeling OR modelling OR fea OR simulation) AND ("poly(l-lactic)" OR "PLA" OR "PLLA" OR polymer)). All of the articles using this query were analysed.

**Radial strength (radially applied load)**

The radial loading parameters have been characterized according to ASTM F3067-14 [1] and ISO 25539-2 [2].

For the radial loading of balloon-expandable stents the radial strength was taken as reported in literature when the value was presented in N/mm. When the value was given in other units, the following relationships has been used:

$${RS}_{L} =\frac{{RS}_{A}}{\pi D}$$

, where RS_L_ is the radial strength calculated from the graph of radial force per length of the stent vs. instantaneous diameter of the stent [N/mm]; RS_A_ – radial strength calculated from the graph of radial force per instantaneous cylindrical area of the stent (i.e., radial pressure) vs. instantaneous diameter of the stent [N/mm^2^]; D – instantaneous diameter of the stent [mm].

Therefore, the radial load on the graphs can be represented by:

$${RL}_{L}=\frac{F_{R}}{L}$$

$${RL}_{A}=\frac{F_{R}}{A}$$

$$A=\pi D L$$

, where RL_L_ is radial load calculated per length of the stent [N/mm]; RL_A_ – radial load calculated per instantaneous cylindrical area of the stent (i.e., radial pressure) [N/mm^2^]; F_R_ - radial force [N]; A – instantaneous cylindrical area of the stent [mm^2^]; L – length of the stent [mm].

RS_L_ [N/mm] and RS_A_ [N/mm^2^] are calculated from the graphs of RL_L_ [N/mm] or RL_A_ [N/mm^2^] vs D [mm], respectively, and correspond to the maximum load for a clinically important permanent deformation.

In case of the use of F_R_ [N] value, as in [3], it was converted to RS_L_ [N/mm] and considered corresponding to the radial strength RS_L_.

The radial displacement was converted to D as follows, except for [4] where the radial displacement was considered a diameter change rather than a radial change:

$$D= D_{0}-2 RD$$

, where D is the instantaneous diameter of the stent [mm]; D_0_ – initial diameter [mm]; RD – radial displacement [mm].

In the case when these graphs were presented without the calculation of radial strength (RS_L_ or RS_A_), as in [4], the radial strength was taken as the maximum on the curve “RL_L_ vs D” after conversion to these axes.

**Chronic outward force (radially applied load)**

The chronic outward force (COF) [N/mm] for self-expanding stents was calculated according to ASTM F3067-14 and ISO 25539-2 [2] as the intersections of the radial load per length RL_L_ [N/mm] vs instantaneous diameter D [mm] curve with two lines: the minimum intended use diameter (i.e., maximum COF) and the maximum intended use diameter (i.e., minimum COF). If the minimum and maximum intended use diameters were not specified, as in [5], they were taken from available product data sheets.

If the COF was not calculated, and the graph was presented as RL_L_ vs change in the cross-sectional area (strain) [6] it was converted to RL_L_ vs D as follows and intended use diameters were taken from available data:

$$D=D_{0}\cdot\frac{strain}{100\%}$$

**Compression or crush resistance (perpendicularly applied load)**

Compression or crush resistance was calculated in the parallel plates compression test at 50% compression:

$$CS=\frac{F_{C}}{L}$$

, where CS is the compression strength [N/mm]; F_C_ – radial force [N]; L – length of the stent [mm].

Compression was determined according to the formula:

$$C=100\%-\left( \frac{D_{p}}{D_{0}}\cdot100\% \right)$$

, where C – compression [%]; D_p_ – instantaneous distance between the plates [mm]; D_0_ – initial diameter [mm].

If compression resistance was unavailable for 50%, the value for the closest compression was given.

**Bending stiffness**

The flexural parameters have been characterized according to ASTM F2606-08 [8].

The bending stiffness was compared using variable span lengths:

$$EI=\frac{PL^{3}}{48\delta}$$

, where EI is the bending stiffness [N∙mm^2^], P – bending load [N], L – span length [mm], δ – deflection [mm].

References

[1] “ASTM F3067-14 Guide for Radial Loading of Balloon Expandable and Self Expanding,” no. March, pp. 1–19, 2015, doi: 10.1520/F3067.

[2] “ISO 25539-2 Cardiovascular implants — Endovascular devices — Part 2: Vascular stents,” 2018.

[3] N. Ni *et al.*, “3D printed peripheral vascular stents based on degradable poly(trimethylene carbonate-b-(L-lactide-ran-glycolide)) terpolymer,” *Polym. Adv. Technol.*, vol. 34, no. 5, pp. 1739–1751, 2023, doi: 10.1002/pat.6007.

[4] Z. Mahmud, A. Nasrin, M. Hassan, and V. G. Gomes, “<scp>3D‐printed polymer</scp> nanocomposites with carbon quantum dots for enhanced properties and in situ monitoring of cardiovascular stents,” *Polym. Adv. Technol.*, vol. 33, no. 3, pp. 980–990, Mar. 2022, doi: 10.1002/pat.5572.

[5] R. Ubachs, O. van der Sluis, S. Smith, and J. Mertens, “Computational modeling of braided venous stents — Effect of design features and device-tissue interaction on stent performance,” *J. Mech. Behav. Biomed. Mater.*, vol. 142, no. January, p. 105857, 2023, doi: 10.1016/j.jmbbm.2023.105857.

[6] K. Maleckis *et al.*, “Comparison of femoropopliteal artery stents under axial and radial compression, axial tension, bending, and torsion deformations,” *J. Mech. Behav. Biomed. Mater.*, vol. 75, no. June, pp. 160–168, 2017, doi: 10.1016/j.jmbbm.2017.07.017.

[7] B. Vogt, “Stiffness analysis of reinforced ureteral stents against radial compression: In vitro study,” *Res. Reports Urol.*, vol. 12, pp. 583–591, 2020, doi: 10.2147/RRU.S285031.

[8] “ASTM F2606-08 Standard Guide for Three-Point Bending of Balloon-Expandable Vascular Stents and Stent Systems.”
